# Supplementary material for: MetaRibo-Seq measures translation in microbiomes
Source: Nat Commun. 2020 Jun 29;11:3268. doi: 10.1038/s41467-020-17081-z (PMC7324362; doi:10.1038/s41467-020-17081-z)
Supplement: Supplementary file 10 — Supplementary Data 7 [file 41467_2020_17081_MOESM10_ESM.zip › File2/Confidence_VeryHigh_Taxonomy/92672_out.krona.html]

Javascript must be enabled to view this page.

members
magnitude
magnitudeUnassigned
count
unassigned
taxon
rank

92672\_out

8

2157
1
superkingdom

phylum
1
28890

class
1
183967

order
1
1235850

1
family
1577788

1915670
species
1

SRS1041031\_contig\_number\_2260

6
superkingdom
2

phylum
6
1239

6
class
186801

6
order
186802

216572
2
family

459786
genus
2

1945593
1
species

SRS1041133\_contig\_number\_21912

1897011
species
1

SRS146813\_contig\_number\_27918


SRS024435\_contig\_number\_27522SRS055017\_contig\_number\_178SRS1041033\_contig\_number\_contig-100\_1990.157528SRS142503\_contig\_number\_53685
species
4
1950914


SRS065397\_contig\_number\_contig-100\_9982.9983
1
